# Interaction between the VP2 protein of deformed wing virus and host snapin protein and its effect on viral replication

**DOI:** 10.3389/fmicb.2023.1096306

**Published:** 2023-02-08

**Authors:** Li Sun, Ming Li, Yueyu Ma, Sichao Huang, Mingxiao Ma, Dongliang Fei

**Affiliations:** ^1^College of Animal Husbandry and Veterinary Medicine, Jinzhou Medical University, Jinzhou, China; ^2^Department of Microbiology, Harbin Medical University, Harbin, China

**Keywords:** deformed wing virus, VP2 protein, snapin, interaction, viral replication

## Abstract

**Introduction:**

Deformed wing virus (DWV) is one of the causative agents of colony collapse disorder. The structural protein of DWV plays a vital role in the process of viral invasion and host infection; however, there is limited research on DWV.

**Methods and Results:**

In this study, we screened the host protein snapin, which can interact with the VP2 protein of DWV, using the yeast two-hybrid system. Through computer simulation and GST pull-down and CO-IP assays, an interaction between snapin and VP2 was confirmed. Furthermore, immunofluorescence and co-localization experiments revealed that VP2 and snapin primarily co-localized in the cytoplasm. Consequently, RNAi was used to interfere with the expression of snapin in worker bees to examine the replication of DWV after the interference. After silencing of snapin, the replication of DWV in worker bees was significantly downregulated. Hence, we speculated that snapin was associated with DWV infection and involved in at least one stage of the viral life cycle. Finally, we used an online server to predict the interaction domains between VP2 and snapin, and the results indicate that the interaction domain of VP2 was approximately located at 56–90, 136–145, 184–190, and 239–242 aa and the snapin interaction domain was approximately located at 31–54 and 115–136 aa.

**Conclusion:**

This research confirmed that DWV VP2 protein could interacts with the snapin of host protein, which provides a theoretical basis for further investigation of its pathogenesis and development of targeted therapeutic drugs.

## Introduction

Honeybees play a significant role as a pollinating insect in food security, agricultural production, and maintenance of natural plant diversity (Klein et al., 2007). In the past 15 years, a sharp decline in the number of honeybee colonies during winter has been reported in different parts of the world (Carreck and Neumann, 2010; Breeze et al., 2014). Decline in the number of pollinators affects beekeeping and agriculture, thereby threatening human food safety (Francis et al., 2013). The main reasons for the decline in the number of bee colonies include pesticide use, pathogens, industries, agriculture, climate change, habitat destruction, and inadequate food supply (Ullah et al., 2021). Viral infections are the key risk factors for the health of honeybees at the individual and colony levels (Ullah et al., 2021). At present, there are at least 24 viruses that can harm the health of bees (Runckel et al., 2011; Gisder and Genersch, 2015, 2017; Dolezal et al., 2016; Remnant et al., 2017; Chagas et al., 2019), among which, the deformed wing virus (DWV) is a major pathogen that is detected at all developmental stages of honeybees and causes colony collapse disorder (De Miranda and Genersch, 2010).

Deformed wing virus is a single-stranded RNA virus belonging to the family *Iflaviridae* and can infect honeybees and other insects (Diao et al., 2019). Its genome is approximately 10,000 bp long with a large open reading frame and encodes a polyprotein of 2,894 amino acids. The polyproteins include structural proteins and nonstructural proteins. The structural proteins consist of VP1, VP2, VP3, and VP4, of which VP1 is 44 kDa in size and is the largest capsid protein of all viruses in the order Picornavirales (Organtini et al., 2016). The size of VP2 (32 kDa) and VP3 (28 kDa) is similar to that of other picornavirus capsid proteins, and VP2 has been reported to exhibit good immunogenicity (Fei et al., 2020). Previous studies have shown that the structural proteins of Iflavirus are involved in viral invasion and replication (Brutscher et al., 2016; Kalynych et al., 2016; Procházková et al., 2018; Zhang et al., 2019). We speculated that the structural proteins of DWV also exert a similar effect. However, only a few studies have explored the function and mechanism of the structural proteins of DWV.

Therefore, in this study, we screened the host proteins that interact with the VP2 protein of DWV using the yeast two-hybrid membrane library system and selected the protein snapin for further investigation. The interaction between snapin and VP2 was determined through Co-IP, GST pull-down assay, immunofluorescence staining, and confocal microscopy. We performed silencing experiments to examine the function of snapin in the process of viral infection. Finally, an online software^1^ was used to predict the interaction domain between VP2 and snapin.

## Materials and methods

### cDNA library, viruses, and primary reagents

The plasmids pOST1-NubI, pTT5, pPR3N, pET28a, pGEX-6P-1, pTSU2-APP, and pBT3STE were procured from the Laboratory Animal Center of Jinzhou Medical University. DWV *VP2*, purified DWV, NMY32 yeast, and *Apis cerana* larvae yeast cDNA libraries were prepared in our laboratory. *Escherichia coli* DH5α and BL21 strains were purchased from TransGen Biotech (Beijing, China). The transfection reagent Lipofectamine 2000 was purchased from GeneCopoeia (Rockville, MD, United States). GST-tagged protein purification kit was purchased from Beyotime (Shanghai, China). Anti-his tag mouse monoclonal antibody was purchased from Solarbio (Beijing, China). The worker bees is provided by the Experimental Animal Center of Jinzhou Medical University.

### Plasmid construction

Total RNA was extracted from worker bees infected with DWV using Trizol reagent (TransGen, Beijing, China) and reverse-transcribed into cDNA using the first-strand cDNA synthesis kit (TransGen). Five pairs of primers were designed for pET28a-VP2, pBT3STE-VP2, pGEX-6p-1-snapin, pTT5-VP2-His, and pTT5-snapin-Flag (Supplementary Table S1). Insertion of enzyme-cutting sites was based on the DWV sequence (GenBank No. MF770715) and snapin gene (GenBank No. XM_006619914). PCR was performed under the following conditions: initial denaturation at 94°C for 5 min, 94°C for 45 s, 58°C for 45 s (pBT3STE-VP2, pET28a-VP2, and pTT5-VP2-His) or 65°C for 45 s (pTT5-snapin-Flag) or 61°C for 30 s (pGEX-6p-1-snapin), 72°C for 45 s, amplification for 30 cycles, and extension at 72°C for 7 min. The PCR products were authenticated using 1.2% agarose gel electrophoresis, purified, and then cloned into vectors. These recombinant vectors were transformed into *E. coli* DH5α and verified through enzyme digestion and sequencing (Genewiz Biological Technology Co., Ltd., Suzhou, China).

### Self-autoactivation and function test

To test the bait plasmid pBT3STE-VP2 with respect to its self-activation and function, it was co-transformed with pPR3N into the reporter NMY32 yeast strain. The co-transformants were grown on SD/−Trp/−Leu, SD/−Trp/−Leu/-His, and SD/−Trp/−Leu/-His/Ala agar plates at 30°C for 3–5 days (Supplementary Table S2).

### Yeast two-hybrid screening

After ensuring that the bait is functional in the membrane protein yeast two-hybrid system, the host cell proteins that interact with VP2 can be screened. We transformed the *A. cerana* larvae cDNA library and the bait plasmid pBT3STE-VP2 into NMY32 cells and cultured them at 30°C for 3–4 days. Then, the NMY32 cells were plated on a series of selective agar plates, including SD/−Trp/−Leu and 60 mM 3-amino-1,2,4-triazole SD/−Trp/−Leu/−Ade/-His selective media, and cultured for 1–2 days at 30°C to select potentially positive colonies. The selected positive colonies were cultured in SD/−Trp/−Leu liquid medium at 30°C for 14 h. Plasmids were extracted from yeast and retransformed into *E. coli* DH5α for subsequent analysis. The plasmids were sequenced by Synbio Technologies Co. Ltd (Jiangsu, China). The resulting sequences were analyzed and aligned using BLAST in NCBI and GenBank databases.

### Molecular docking of VP2 with snapin

Because there was no experimental structure available for snapin, it was modeled using the I-TASSER server^2^ and modified using the GalaxyRefine server.^3^

In contrast, since an experimental structure was available for VP2, it was modeled using the SWISS-MODEL workspace.^4^

The ClusPro server^5^ is a widely used tool for protein–protein docking and can be used to predict protein interactions. To reveal the binding affinity between VP2 and snapin, we used the ClusPro server for docking analysis.^6^ Snapin acts as a receptor for antigen recognition. The ClusPro server computed the models based on desolvation energy and electrostatic interactions.

### GST pull-down assay

The GST pull-down assay was performed according to the manufacturer’s instructions (Bio-Works, Sweden) to confirm the interaction detected between snapin and VP2. The plasmids PGEX-6p-1-snapin and PET28-VP2 were transformed into *E. coli* BL21 (DE3) for induction, expression, and purification. The purified GST–snapin fusion protein was incubated with glutathione agarose beads at 4°C for 3 h, followed by overnight incubation at 4°C with 0.1 mg/ml of the input protein His-VP2. The agarose complex was eluted, separated, collected, centrifuged, and solubilized in 2× sodium dodecyl sulfate sample buffer. Next, western blotting was performed to analyze the collected proteins. As a negative control, GST was incubated with beads alone with *E. coli* lysates.

### Co-IP assays

Co-IP assays were performed to further confirm the interaction between snapin and VP2 in cells. pTT5-VP2-His and pTT5-snapin-Flag were co-transfected into BHK cells. After 48 h, the cell lysates were harvested by centrifugation (Thermo Fisher, Waltham, MA, United States) at 12,000 rpm for 25 min at 4°C and incubated overnight with a Flag-tagged antibody (Proteintech, Wuhan, China) at 4°C. Then, Protein A/G Plus-Agarose (Santa Cruz Biotechnology, Santa Cruz, CA, United States) was added, and the mixture was incubated at 4°C for 6 h. This was followed by centrifugation at 1500 rpm for 3 min at 4°C, and the resulting precipitate was washed three times with ice-cold PBS and finally identified using western blotting.

### Detection of co-localization *via* immunofluorescence staining and confocal microscopy

The plasmids pTT5-VP2-His and pTT5-snapin-Flag were co-transfected into BHK cells. After 24 h, the cells were fixed in 2 ml of 4% paraformaldehyde (Solarbio, Beijing, China) at 4°C for 20 min and then washed three times with PBS. Next, the cells were permeabilized with 2 ml of 0.5% Triton X-100 (Sangon Biotech, Shanghai, China) for 30 min at room temperature, washed three times with PBS, and then blocked in Tris-buffered saline-Tween 20 (TBST) containing 2% bovine serum albumin (Solarbio) for 1 h at room temperature. The coverslips were incubated overnight together with His-tagged and Flag-tagged antibodies (1:400 dilution; Proteintech) at 4°C. The cells were washed three times with PBS. The coverslips were then incubated with goat anti-mouse and goat anti-rabbit fluorescent antibodies (diluted 1:2000 with TBST) in the dark for 1 h at 37°C. DAPI (Coolaber, Beijing, China) (1:750 dilution) was used to stain the cell nucleus in the dark for 20 min, after which the stained cells were viewed under a laser scanning confocal microscope (LEICA, Germany).

### Snapin level in honeybees after deformed wing virus infection

A total of 20 healthy worker bees were selected from the same colony and randomly divided into two groups, with each group containing 10 bees. The bees were placed on ice and anesthetized with CO_2_ for 1 min. Group I was injected with 1 μL of a DWV suspension between the 2nd and 3rd integuments using a similar controlled-volume syringe (Huang et al., 2020), and group II was injected with 1 μL PBS as control. The inoculated bees were fed with sufficient sterilized food and water. The clinical signs in each group of bees were monitored and recorded daily until the bees died. RT-PCR was performed to detect the infection of DWV, Chinese sacbrood virus (CSBV), Chronic bee paralysis virus (CBPV), Black queen cell virus (BQCV), Kashmir bee virus (KBV), Israel acute paralysis virus (IAPV), and Acute Bee Paralysis Virus (ABPV) in dead bees (Hassanyar et al., 2019). The levels of snapin in bees after DWV infection were analyzed *via* SYBR Green RT-PCR.

### Effect of silencing of snapin on VP2 expression

We conducted silencing experiments to examine the function of snapin in the process of viral infection. Three pairs of siRNAs of snapin were designed and synthesized by Suzhou Jima Biotechnology Co., Ltd. A total of 40 healthy worker bees were retrieved from the same colony and randomly distributed into four groups (groups 1–4), with each group containing 10 bees. Group 1 was injected with 1 μg of siRNA1, group 2 was injected with 1 μg of siRNA2, group 3 was injected with 1 μg of siRNA3, and group 4 was injected with 1 μL PBS. After 72 h, three bees were randomly selected from each group for qPCR analysis.

After screening the best siRNA capable of inhibiting the function of snapin, 20 worker bees were retrieved from the same colony and randomly distributed into two groups (groups 5 and 6), with each group containing 10 bees. Group 5 was injected with 1 μg of siRNA and group 6 was injected with 1 μL of PBS. After 24 h, groups 5 and 6 were injected with 1 μL of DWV. After 72 h, three bees were randomly selected from each group, and the replication of DWV was analyzed *via* qPCR (Bradford et al., 2017).

### Predictive analysis of the interaction pocket

The docking model was analyzed using the PDBePISA server (see text footnote 1) and according to the interface area Å2 and ΔIg kcal/mol to judge the docking effect. The interaction domain between VP2 and snapin was analyzed according to the interactions between the protein chains and residual interactions across the interface using the PDBsum server.^7^

## Results

### Construction of recombinant plasmids

**Figure 1:**
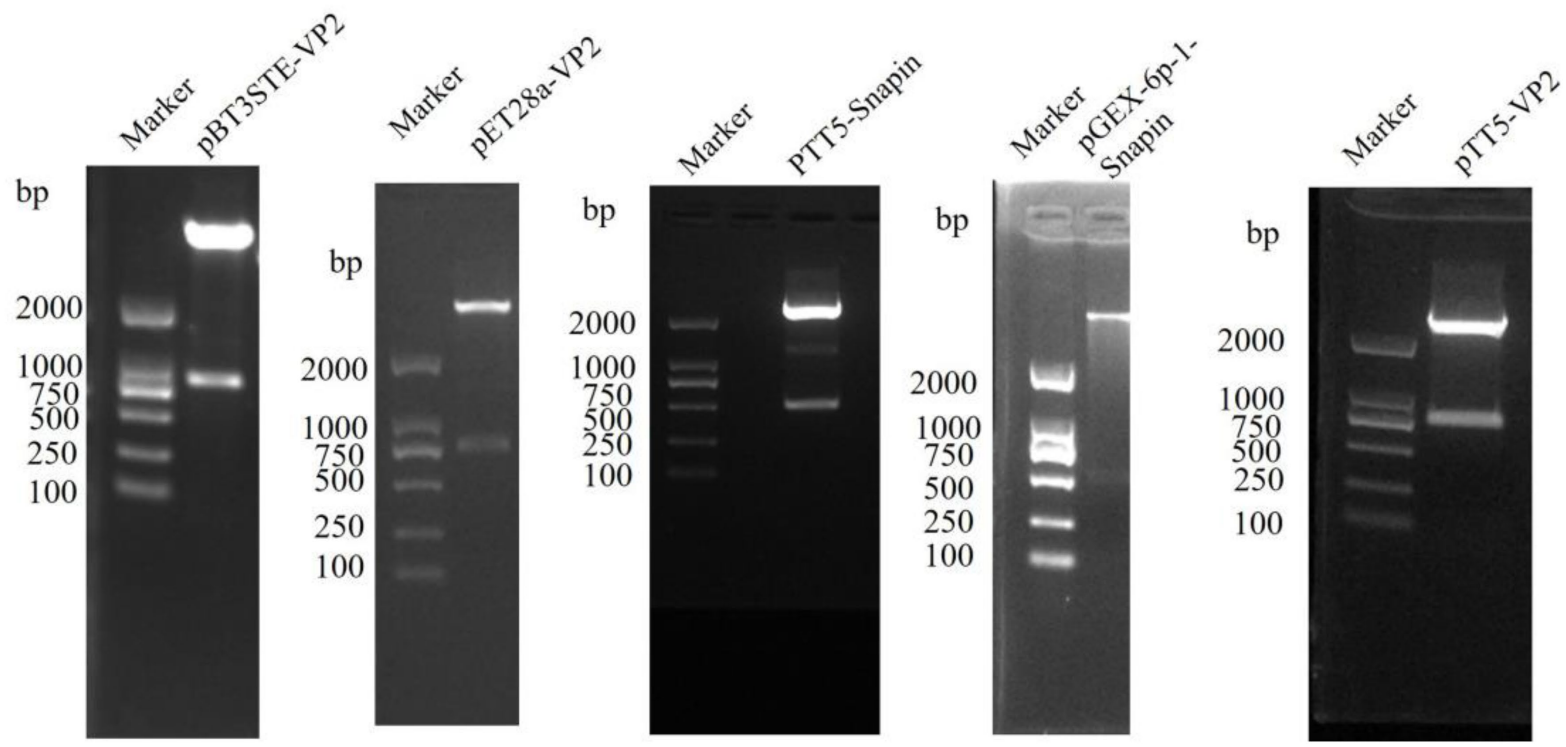
Restriction endonuclease digestion results of construction plasmid of pBT3STE-VP2, pET28a-VP2, pTT5-Snapin-Flag, pGEX-6p-1-Snapin, and pTT5-VP2-His.

The positive recombinant plasmids pBT3STE-VP2, pET28a-VP2, pGEX-6p-1-snapin, pTT5-snapin-Flag, and pTT5-VP2-His were identified through restriction digestion and sequencing analysis, which indicated that all recombinant plasmids were successfully constructed (Figure 1).

### Autoactivation and function detection of the pBT3STE-VP2 plasmid

Selective plate assays revealed that white clones grew on all SD/−Trp/−Leu plates. In the positive control and functional test groups, white clones were detected on the solid plates of SD/−Trp/−Leu/-His and SD/−Trp/−Leu/−Ade/-His; however, in the self-activation detection and negative control groups, no such clones were observed (Supplementary Figure S1). These results show that the bait plasmid pBT3STE-VP2 had function and no autoactivation on Y2H. It was used for subsequent screening experiments.

### VP2 Y2H screening

The yeast two-hybrid system was used to screen for VP2-interacting host proteins from the *A. cerana* cDNA library. A total of 24 possible yeast proteins with a positive bait–prey interaction were screened from the membrane protein Y2H system and their reporter gene was detected. Sequence analysis of the 24 positive plasmids indicated that they represented 20 *A. cerana* cDNAs of potential protein genes interacting with VP2 (Supplementary Table S3). After analysis, we believe that the snapin gene is a useful host protein in these genes. Therefore, we selected the snapin gene for further investment.

### Prediction, refinement, and docking prediction of snapin and VP2 tertiary structures

**Figure 2:**
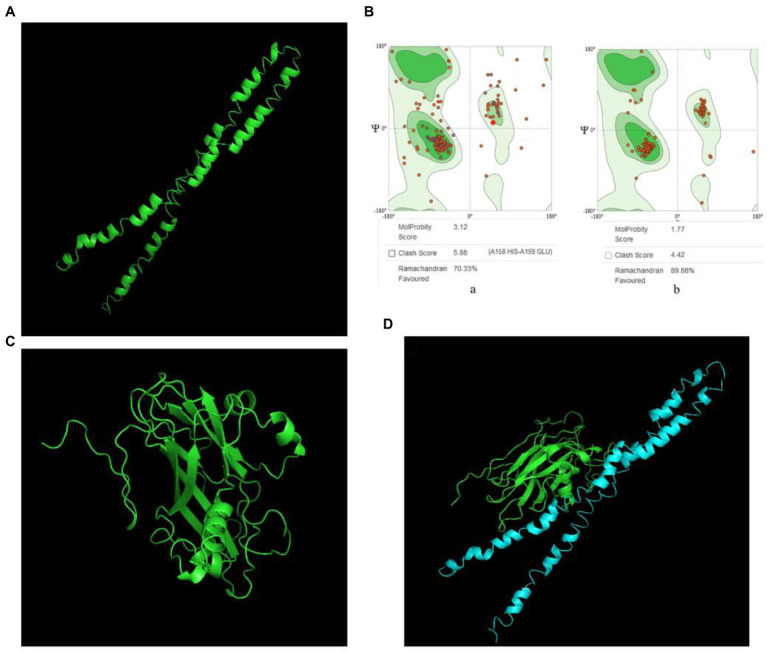
VP2 and snapin interaction prediction. **(A)** Optimized snapin 3D model. **(B)** Ramachandran plots. **a**, snapin model modeled using the I-TASSER SERVER; **b**, optimized snapin model. **(C)** 3D model of VP2 protein. **(D)** Molecular docking model of VP2 and snapin. The green model is VP2 and the blue model is snapin.

Snapin was modeled using the I-TASSER server and five models were generated. Model 1 had the top Ramachandran favored and was therefore selected for optimization. The initial model 1 was optimized in the GalaxyRefine server that generated five models based on repeated structure perturbation and subsequent overall structural relaxation *via* molecular dynamics simulation. Based on the comprehensive analysis of TM score, root mean square deviation (RMSD), MolProbity, and Ramachandran plot, model 1 was selected for docking purposes (Figure 2A). The initial model 1 generated from the I-TASSER server and the refined model 1 generated from the GalaxyRefine server were evaluated using the SWISS-MODEL workspace. The initial model 1 and the refined model 1 had 70.33 and 89.88% of residues in the Ramachandran favored region, respectively (Figure 2B). Other favorable parameters of the refined model were as follows: GDT score of 0.8926, RMSD value of 0.554, MolProbability of 1.983, clash score of 7.9, and poor rotamers totaling 0.6 (Supplementary Figure S2).

VP2 was modeled using the SWISS-MODEL workspace and two models were generated. Model 1 had the top Global Model Quality Estimate and Ramachandran favored and was therefore selected for docking purposes (Figure 2C).

The ClusPro server yielded 30 candidate models with different binding energies, among which, model complex 1 with the lowest binding energy score of −1011.6 was chosen (Figure 2D). The interaction between VP2 and snapin was predicted.

### GST pull-down assay

**Figure 3:**
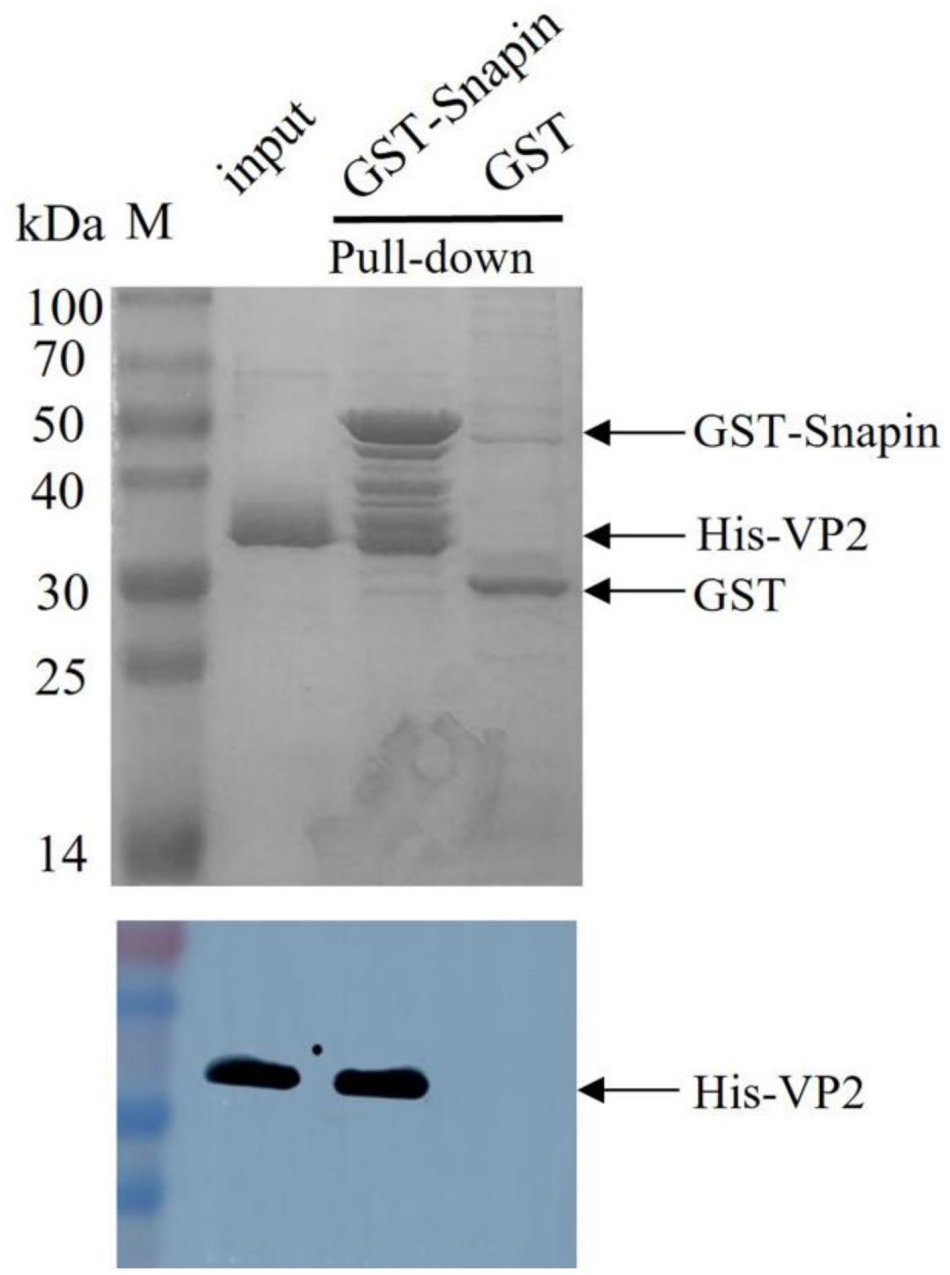
SDS-PAGE (12%; Coomassie blue staining; top) and Western blotting analysis (bottom) of GST pull-down samples. GST tag was used as the negative control, His-VP2 was used as the positive control, GST-snapin was used as the experimental group. Compared with the negative control group, the experimental and positive control groups showed a protein band with a molecular weight of approximately 33 kDa, which was consistent with the expected VP2 protein sizes (top), indicating that VP2 and GST-snapin may have formed a complex. Western blotting was performed with anti-His antibody. His-VP2 protein appeared at a molecular mass of approximately 33 kDa (bottom).

The GST pull-down assay was performed to detect the interaction between VP2 and host snapin *in vitro*. In this experiment, GST–snapin was used as the bait protein, His-VP2 was used as the prey protein, and GST tag was used as the negative control. The results were analyzed *via* western blotting. The experimental and positive control groups exhibited an approximately 33-kDa protein band, consistent with the expected His-labeled VP2 protein sizes, whereas the negative control group exhibited no protein band (Figure 3). These results indicated the presence of an interaction between VP2 and snapin.

### Co-IP assay

**Figure 4:**
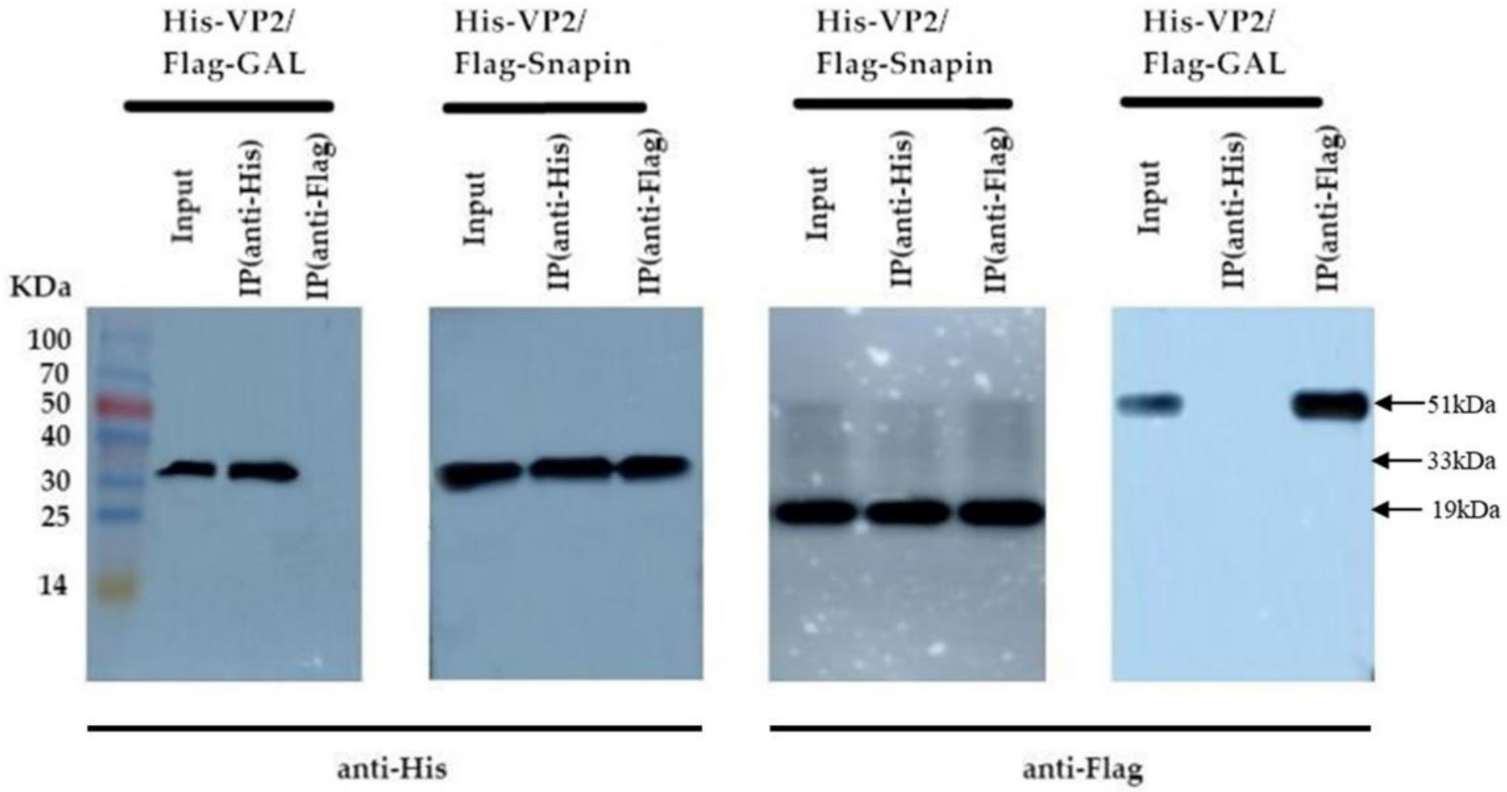
Co-IP assay results of the interaction between VP2 and snapin. The co-transformed pTT5-VP2-His and Gal-Flag recombinant plasmids were used as the negative control. In the recovered products, VP2 protein bands were detected using anti-His antibody, and snapin protein bands were detected using anti-Flag antibody. No corresponding band was detected in the negative control.

Co-IP assay is a common technique to identify or confirm physiologically relevant protein–protein interaction events and can be used for further confirmation of the interaction between VP2 and snapin. We performed Co-IP assay in BHK cells coexpressing snapin-Flag and VP2-His to further determine whether the interaction between snapin and VP2 occurs *in vitro*. As shown in Figure 4, detection with either anti-Flag or anti-His antibodies revealed the corresponding bands, demonstrating a specific interaction between snapin and VP2 in the cells.

### Detection of co-localization *via* immunofluorescence staining and confocal microscopy

**Figure 5:**
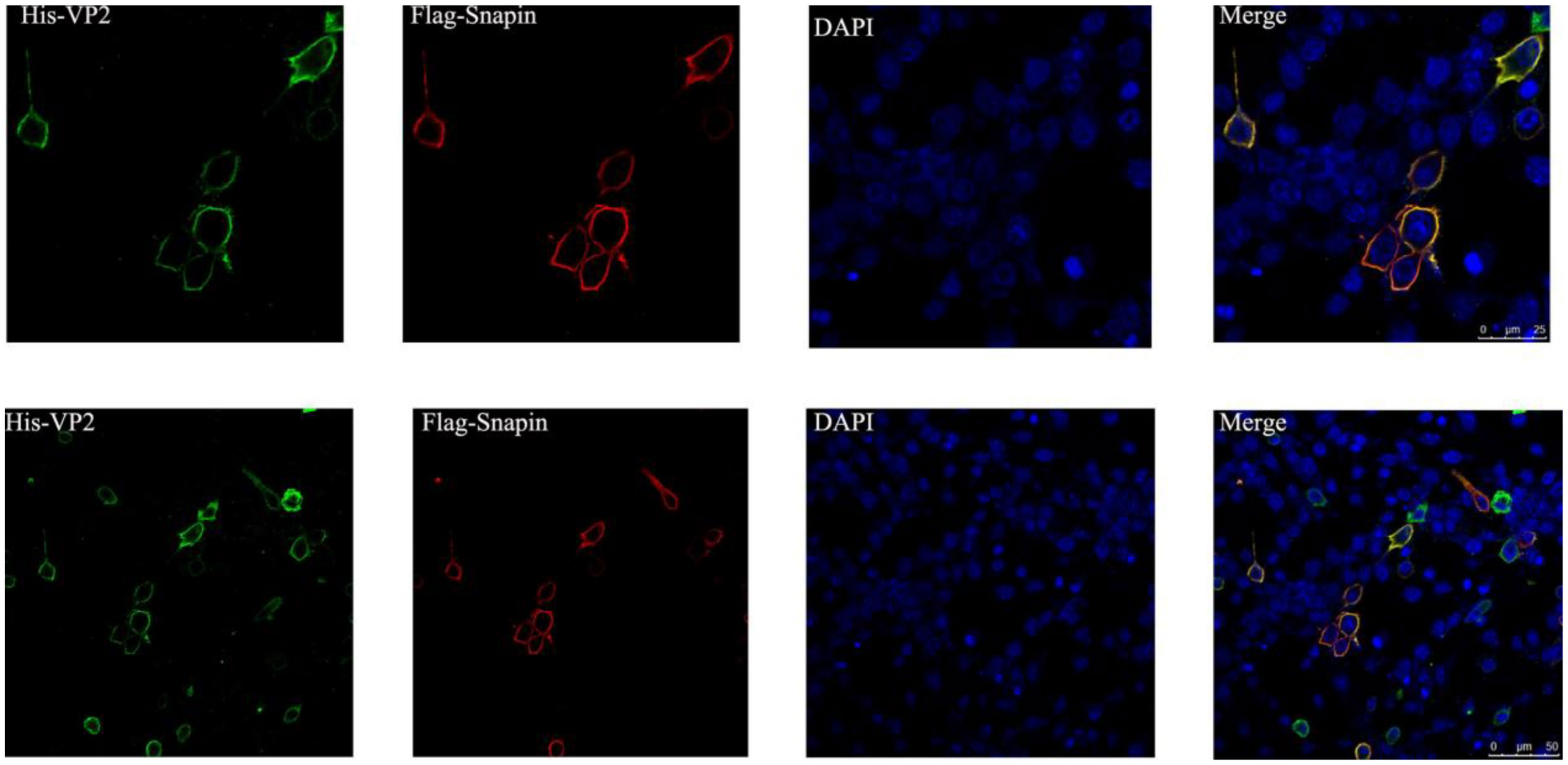
Immunofluorescence staining and confocal microscopy results showing the co-localization of VP2 and snapin. Red, snapin; green, VP2; and blue, nucleus. Co-localization of VP2 and snapin is indicated in yellow in the merged image.

To determine whether VP2 and snapin localize within the same cellular compartment, the recombinant plasmids pEGFP-snapin-Flag and pTT5-VP2-His were co-transfected into BHK cells. As shown in Figure 5, both snapin and VP2 localized in the cytoplasm. The results of co-localization revealed that VP2 and snapin could completely overlap in cells and were primarily localized in the cytoplasm.

### Snapin level in honeybees after deformed wing virus infection

**Figure 6:**
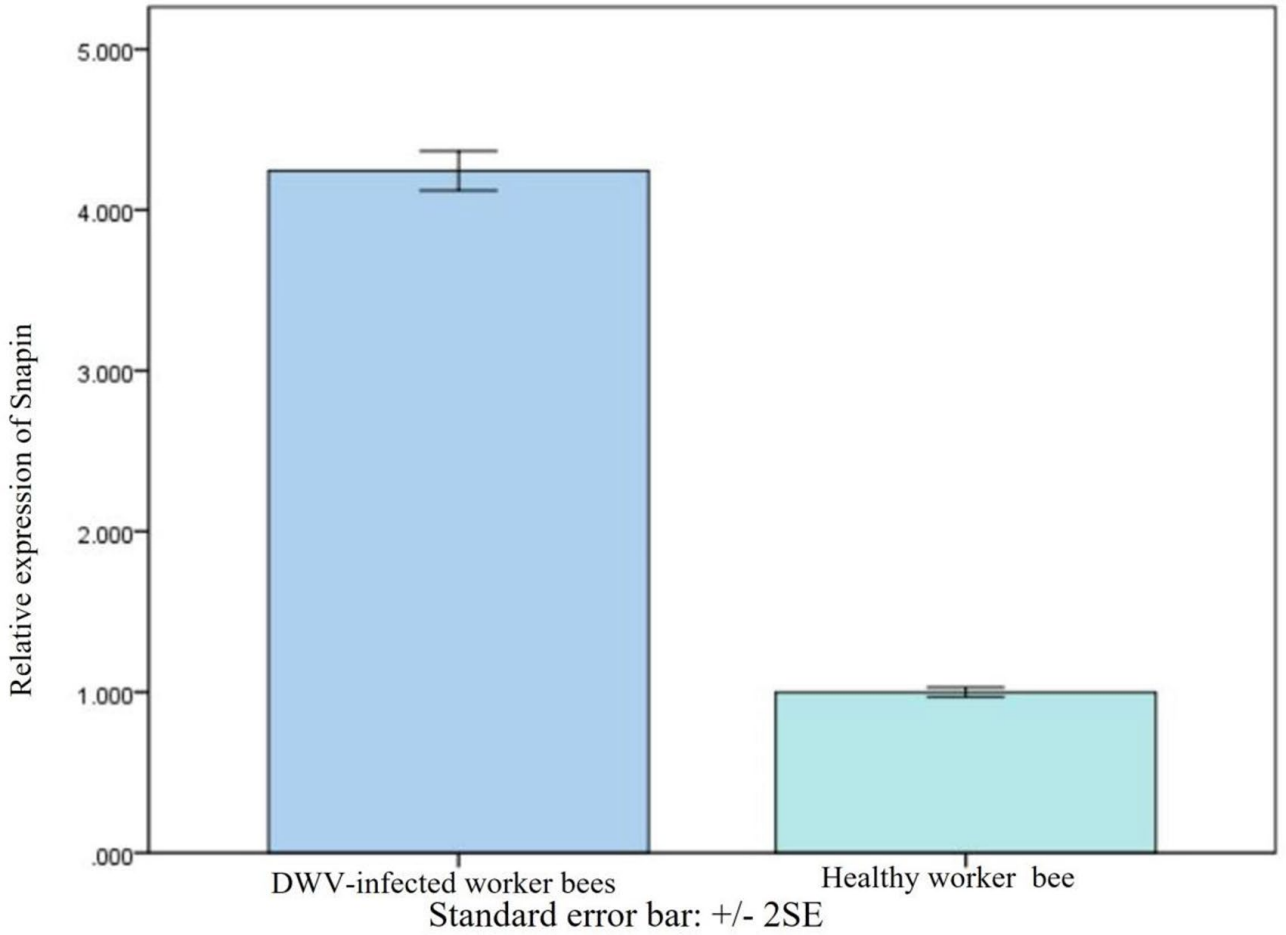
Comparison of snapin levels between healthy and DWV-infected worker bees. The level of snapin increased after the worker bees were infected with DWV. Error bar represents standard error.

We conducted SYBR Green RT-PCR to further investigate the changes in snapin expression in worker honeybees after DWV infection. Results indicated that the expression of snapin in worker honeybees was increased after DWV infection (Figure 6). Moreover, all honey bees used during the experiments were analyzed using RT-PCR, and the results showed that no honey bee viruses were detected in healthy workers and that only DWV was detected in infected workers (Supplementary Figure S3).

### Effect of silencing of snapin on VP2 expression

**Figure 7:**
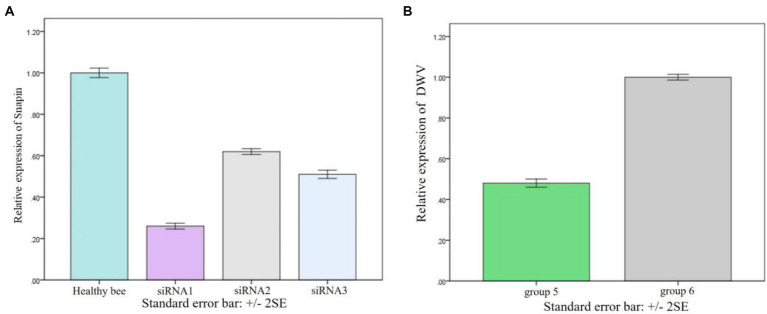
Result of snapin silence. **(A)** Changes in snapin level after siRNA silencing. The expression level of snapin in healthy bees was used as the standard control. Error bar represents standard error. **(B)** Replication of DWV in worker bees after silencing of snapin with siRNA. Group 6 was used as the control group, and the replication of DWV was significantly downregulated after silencing of snapin with siRNA in group 5. Error bar represents standard error.

We have found that snapin expression was upregulated *in vivo* when the worker bees were infected with DWV. To examine the function of snapin in the process of DWV infection, we conducted silencing experiments. Compared with other siRNAs, siRNA1 exerted the maximum inhibitory effect on snapin expression (Figure 7A). Therefore, we selected siRNA1 for subsequent experiments. Results showed that DWV expression was significantly downregulated in group 5 (Figure 7B), indicating that snapin was involved in the replication of DWV in honeybees.

### Analysis of molecular docking between VP2 and snapin

**Figure 8:**
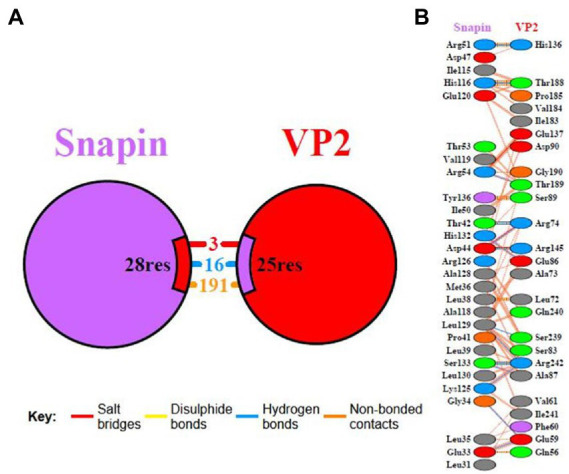
Interaction domain analysis between VP2 and snapin. Interacting chains are joined by colored lines, each representing a different type of interaction. **(A)** The area of each circle is proportional to the surface area of the corresponding protein chain. The extent of the interface region on each chain is represented by the black wedge whose size signifies the interface surface area. **(B)** Statistics for this interface are given.

The interaction domain between VP2 and snapin was analyzed using the PDBsum server, and the results are shown in Figure 8. We detected 3 salt bridges and 16 hydrogen bonds in the interaction between VP2 and snapin at the protein–protein interface. The protein–protein interaction site of VP2 was approximately located at 56–90, 136–145, 184–190, and 239–242 aa. The protein–protein interaction site of snapin was approximately located at 31–54 and 115–136 aa.

## Discussion

Honeybees are important pollinators; however, there has been a severe decline in bee populations in the past 20 years. The main reason for the serious decline in honeybee colonies is the interaction between parasites and pathogens, including viruses, mites, fungi, bacteria, and other pests (Goulson et al., 2015). Among several diseases caused by pathogens, viral diseases are considered to be the primary threat to apiculture, and more than 20 honeybee viruses have been confirmed to infect honeybees (Berényi et al., 2007; Baker and Schroeder, 2008; Reddy et al., 2013). DWV is a highly prevalent and pathogenic honeybee virus and can cause colony collapse disorder (Organtini et al., 2016; Kevill et al., 2017). Recently, several studies have investigated the structure and genes of DWV (Mordecai et al., 2016; Organtini et al., 2016; Dalmon et al., 2017; Škubník et al., 2017; Tehel et al., 2019); however, information on the mechanism underlying DWV infection is limited.

In this study, we used the yeast two-hybrid system to capture the proteins interacting with the VP2 protein of DWV and found that snapin could interact with VP2. We used the I-TASSER server and SWISS-MODEL server to model VP2 and snapin and selected the best docking optimization model, following which the ClusPro server was used for docking. We found that VP2 and snapin could interact with each other. The GST pull-down assay revealed expected bands in the experimental group, indicating the interaction between VP2 and snapin. The results of the Co-IP assay revealed the presence of a specific interaction between snapin and VP2 in cells. Immunofluorescence staining and confocal microscopy demonstrated that VP2 and snapin were completely overlapped in cells and were primarily localized in the cytoplasm. These results suggest that VP2 can interact with snapin and co-localize with it in the cytoplasm. After DWV infection in worker honeybees, the expression of snapin was significantly upregulated. Silencing of snapin *via* siRNA interference resulted in significant downregulation of DWV replication in the worker honeybees. These results showed that snapin was associated with DWV infection and involved in at least one stage of the viral life cycle.

Snapin was originally detected as a SNAP-25-binding protein in the SNARE complex in nerve cells. It has been reported to be expressed in both the cytosol and plasma membrane of nerve cells and non-nerve cells and is an important component of the neurotransmitter release process (Ilardi et al., 1999). Snapin consists of 136 amino acids, with a relative molecular weight of approximately 15 kDa. It plays a key role in cell vesicle transport and fusion and has been implicated in the regulation of exocytosis and endocytosis (Hertel and Mocarski, 2004). A previous study suggested that snapin serves as an important regulator of the late endocytic fusion machinery (Li et al., 2009). Another study showed that snapin acts as a dynein motor adaptor and coordinates retrograde transport and late endosomal–lysosomal trafficking, thereby maintaining efficient autophagy–lysosomal function in neurons (Cai and Sheng, 2011). It has been reported that the viral proteins pul130, ul70, ul105, and UL142 interact with the host protein snapin and affect viral DNA replication in human cytomegalovirus infection (Shen et al., 2011; Luo et al., 2013; Liu et al., 2015; Wang et al., 2016). After infection in bees, DWV can cause abnormal neural activity and movement. Our study showed that VP2 and snapin were co-localized in the cytoplasm, and the expression of snapin was significantly upregulated after DWV infection in worker bees. Moreover, the replication of DWV was significantly downregulated after snapin silencing. Based on our results, we speculate that snapin participates in the processes of nerve cell autophagy, nerve injury, and incomplete autophagy induced by DWV.

To summarize, the host protein snapin that interacts with VP2 of DWV was screened for the first time and a direct interaction between VP2 and snapin was confirmed *in vitro*. Moreover, VP2 and snapin were found to be highly co-localized in co-transfected BHK cells. The regulatory effect of snapin on DWV replication was confirmed based on the results of worker bee infection and siRNA interference experiments. We speculate that snapin affects the assembly of virus particles in the cytoplasm and participates in the process of nerve cell autophagy induced by DWV. The interaction pocket between VP2 and snapin was predicted, which provided a theoretical basis for further investigation of the pathogenesis of DWV infection and the development of targeted therapeutic drugs.

## Data availability statement

The datasets presented in this study can be found in online repositories. The names of the repository/repositories and accession number(s) can be found in the article/Supplementary material.

## Author contributions

LS and MM designed the study and wrote the manuscript. ML, YM, SH, and DF performed the experiments and analyzed the data. All authors approved the final version of the manuscript.

## Funding

This work was supported by grants from the National Natural Science Foundation of China (Nos. 31972626 and 32172789), Natural Science Fund of the Liaoning Province (No. 2020-MS-300 and 2022-MS-385), Science and Technology Research Project of Educational Department of Liaoning Province (No. LJKMZ20221222), and Horizontal Project of Jinzhou Medical University (Nos. 2021002 and 2021025).

## Conflict of interest

The authors declare that the research was conducted in the absence of any commercial or financial relationships that could be construed as a potential conflict of interest.

## Publisher’s note

All claims expressed in this article are solely those of the authors and do not necessarily represent those of their affiliated organizations, or those of the publisher, the editors and the reviewers. Any product that may be evaluated in this article, or claim that may be made by its manufacturer, is not guaranteed or endorsed by the publisher.

## Footnotes

^1^ https://www.ebi.ac.uk/msd-srv/prot_int/pistart.html

^2^ https://zhanggroup.org/I-TASSER/

^3^ https://galaxy.seoklab.org/cgi-bin/submit.cgi?type=REFINE

^4^ https://swissmodel.expasy.org/assess

^5^ https://cluspro.org

^6^ https://cluspro.bu.edu/login.php?redir/queue.php

^7^ http://www.ebi.ac.uk/
