# Supplementary material for: Interaction between the VP2 protein of deformed wing virus and host snapin protein and its effect on viral replication
Source: Front Microbiol. 2023 Feb 8;14:1096306. doi: 10.3389/fmicb.2023.1096306 (PMC9945523; doi:10.3389/fmicb.2023.1096306)
Supplement: Supplementary file 1 [file Table_1.DOCX]

| Name Primers Sequence (5’ to 3’) Restriction enzyme  sites | | | |
| --- | --- | --- | --- |
| pBT3STE-VP2 | VP2 F | 5’-GCGGCCATTACGGCCATGCCTGATGGCGAGGGA-3’ | SifI |
|  | VP2 R | 5’-GCGGCCGAGGCGGCCTCAGGTTTTGCCCTGAT-3’ | SifI |
| pET28a-VP2 | VP2 F | 5’-GCGAATTCATGCCTGATGGCGAGGGTGAA-3’ | EcoRI |
|  | VP2 R | 5’-GCAAGCTTTTACTCAGGTTTTGCCCTGAT-3’ | HindIII |
| pGEX-6p-1-snapin | snapin F | 5’- GCGGATCCATGgacgtggatacagcaagtgat-3’ | BamHI |
|  | snapin R | 5’- GCGTCGACTAAttcatgttctttagaaatattagt-3’ | SalI |
| pTT5-VP2-His | VP2 F | 5’-GCGAATTCGCCGCCACCATGCCTGATGGCGAGGGTGAAG -3’ | EcoRI |
|  | VP2 R | 5’-GCGGATCCTTAGTGGTGATGGTGGTGGTGGGAACCACCAC CACCCTCAGGTTTTGCCCTGATTT-3’ | BamHI |
| pTT5-snapin-Flag | snapin F | 5’-GCGAATTCGCCGCCACCATGgacgtggatacagcaagtgat -3’ | EcoRI |
|  | snapin R | 5’-GCAAGCTTTTACTTATCGTCGTCATCCTTGTAATCAGAGCCGCCGCCGCttcatgttctttagaaatattagt-3’ | HindIII |
| snapin | snapin F | 5’- catctattttgtgttttgtct -3’ |  |
|  | snapin R | 5’- gattcattcatgttctttag -3’ |  |

**Supplementary Table 1** Primers used in this study

**Supplementary Table 2** Transfer of various plasmid combinations into the recipient strain NMY32.

| **Reaction** | **AD plasmid** | **BD plasmid** | **Coating plate type** | **remarks** |
| --- | --- | --- | --- | --- |
| 1 | pPR3N (NubG- nonsence) | pTSU2-APP (Cub-fused APP) | SD-TL、SD-TLH、SD-TLHA | negative control |
| 2 | pNubG-Fe65(NubG-fused Fe65) | pTSU2-APP (Cub-fused APP) | SD-TL、SD-TLH、SD-TLHA | positive controls |
| 3 | pOST1-NubI(wild-type NubI) | pBT3STE-VP2(Cub-fused VP2) | SD-TL、SD-TLH、SD-TLHA | function test |
| 4 | pPR3N (NubG- nonsence) | pBT3STE-VP2(Cub-fused VP2) | SD-TL、SD-TLH、SD-TLHA | Self-activation detection |

**Supplementary Table 3** Sequence analysis of the positive plasmids

| Positive clone number | Gene Bank | English name |
| --- | --- | --- |
| 07 | NW_016017601.1 | PREDICTED: Apis cerana 60S ribosomal protein L12 |
| 09 | NW_016019375.1 | PREDICTED: Apis cerana uncharacterized |
| 11 | NW_016018712.1 | PREDICTED: Apis cerana glutathione S-transferase-like |
| 12 | NW_016017900.1 | PREDICTED: Apis cerana protein TSSC4 （tumor Suppressing Subtransferable Candidate 4） |
| 27 | NC_037653.1 | PREDICTED: Apis mellifera 40S ribosomal protein S7 |
| 35 | NW_016019120.1 | PREDICTED: Apis cerana anaphase-promoting complex subunit 15-like |
| 37 | NW_016019441.1 | PREDICTED: Apis cerana 40S ribosomal protein S7-like |
| 53,76 | NW_016019763.1 | PREDICTED: Apis cerana uncharacterized LOC108002651 |
| 58 | NW_016019863.1 | PREDICTED: Apis cerana elongation factor 1-alpha |
| 66 | NW_016019441.1 | PREDICTED: Apis cerana 10 kDa heat shock protein, mitochondrial |
| 68 | NW_016018135.1 | PREDICTED: Apis cerana endoribonuclease Dcr-1-like |
| 69 | XM_017063282.2 | PREDICTED: Apis cerana glycine-rich cell wall structural protein |
| 70 | NW_016018566.1 | PREDICTED: Apis cerana heat shock 70 kDa protein cognate 4 |
| 73 | XM_009592047.2 | PREDICTED: Nicotiana tomentosiformis thiamine thiazole synthase, |
| 78 | NW_006263703.1 | PREDICTED: Apis dorsata SNAPIN protein homolog |
| 82 | NW_016017567.1 | PREDICTED: Apis cerana natterin-3-like |
| 93 | NW_016019131.1 | PREDICTED: Apis cerana 3-hydroxyacyl-CoA dehydrogenase type-2 |
| 94 | NW_016017601.1 | PREDICTED: Apis cerana ribonuclease UK114 |
| 95 | NW_016018111.1 | PREDICTED: Apis cerana nascent polypeptide-associated complex subunit alpha |
| 96 | NW_016019441.1 | PREDICTED: Apis cerana ruvB-like 2 |
| 39,74,10 | Not find |  |
