# Supplementary figures and images for: Interaction between the VP2 protein of deformed wing virus and host snapin protein and its effect on viral replication
Source: Front Microbiol. 2023 Feb 8;14:1096306. doi: 10.3389/fmicb.2023.1096306 (PMC9945523; doi:10.3389/fmicb.2023.1096306)

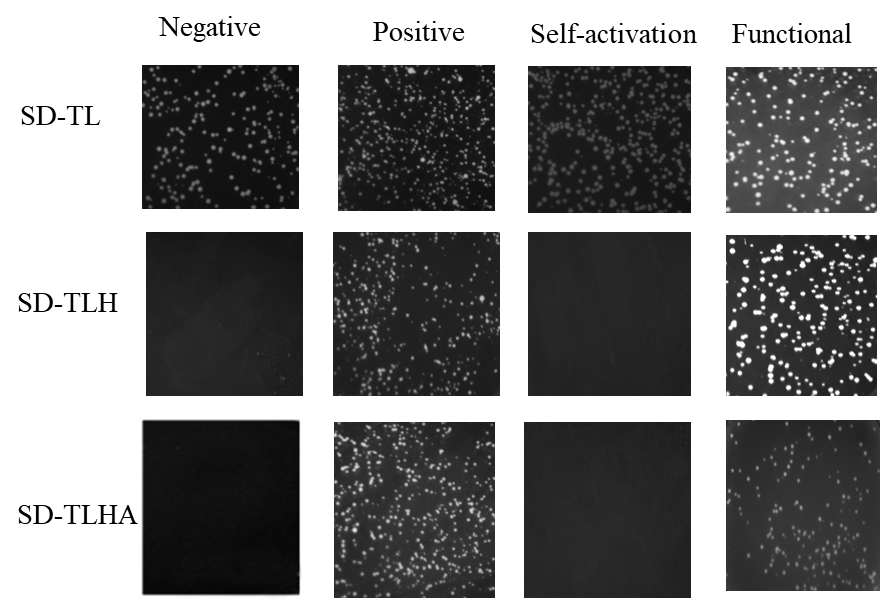

Supplement: Supplementary file 2 [file Image_1.PNG]

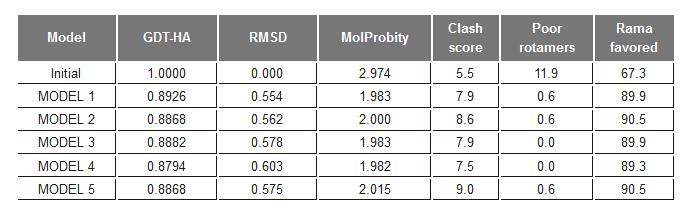

Supplement: Supplementary file 3 [file Image_2.JPEG]

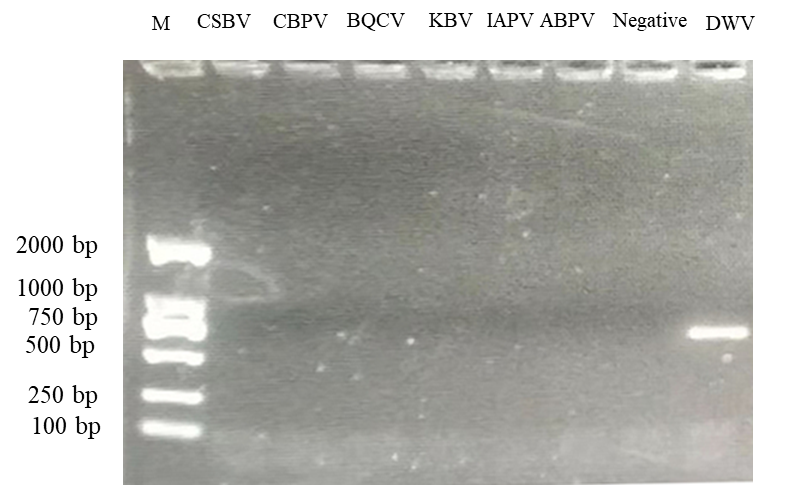

Supplement: Supplementary file 4 [file Image_3.PNG]
